# A systematic review of latent class analysis in psychology: Examining the gap between guidelines and research practice

**DOI:** 10.3758/s13428-025-02812-1

**Published:** 2025-10-03

**Authors:** Angela Sorgente, Rossella Caliciuri, Matteo Robba, Margherita Lanz, Bruno D. Zumbo

**Affiliations:** 1 Department of Psychology, Università Cattolica del Sacro Cuore, Largo Gemelli, 1–20123, Milan, MI Italy; 2 University of British Colombia, Vancouver, Canada

**Keywords:** Latent class analysis, Psychology, Systematic review, Tutorials, Guidelines

## Abstract

Latent class analysis (LCA) can help identify unobserved classes of individuals in a population based on collected categorical data. It is commonly used in psychology to test hypotheses about sources of heterogeneity and class characteristics. However, careful decision-making is required in the modeling process. Its flexibility may explain why it is becoming more commonly used in psychology; however, it also highlights that there are many decision points in the modeling process, thus warranting a systematic literature review to document the use of LCA in psychology, mapping both the prevalence and quality of LCA studies. This systematic review followed the PRISMA guidelines and involved a comprehensive search across multiple databases, yielding 7,580 records related to latent class analysis. After removing duplicates and selecting a representative subsample, 377 documents were assessed for eligibility. Of these, 251 publications (comprising 313 LCAs) met the inclusion and exclusion criteria and were reviewed for this study. Each study was meticulously coded to map how the authors performed and reported each step of the LCA. Our analysis of these studies, in comparison with published guidelines, revealed notable discrepancies in how LCA is applied and reported. To support researchers in enhancing the quality of future LCA applications, we summarize key recommendations in a final section that outlines best practices for future LCA applications. The findings indicate a growing use of LCA in psychology but also highlight the need for greater methodological rigor and transparency in its implementation.

The concept of latent (unobserved) subpopulations is a powerful tool in contemporary multivariate analysis. It helps us identify and understand diverse groups within a larger population. This understanding enriches our knowledge of the population by revealing the diversity within it, and it also provides a more accurate description of the relationships among the observed variables in the data. When we are hesitant to assume homogeneity, we can consider potential heterogeneity using a multivariate method called latent class analysis (LCA), which refers to the unobserved groups of individuals as latent classes. LCA allows us to gain a greater understanding by challenging the assumption that the relationships among the observed variables are the same for all individuals in a population and instead recognizing that relationships can vary across subpopulations. In short, LCA is a multivariate method for analyzing the relationships among manifest data when some variables are unobserved and represent latent classes. The unobserved variables are categorical, allowing the original dataset to be segmented into a number of mutually exclusive and exhaustive subsets: the latent classes.

LCA is a statistical method that belongs to the family of latent variable mixture modeling approaches that aim to find heterogeneity within a population (Petersen et al., 2019). LCA is a type of latent variable model-based method that assumes that the categorical latent variable is responsible for population heterogeneity and that this categorical latent variable aims to describe the underlying mean and covariance structure of observed data (Collins & Lanza, 2010). LCA makes an assumption of local independence in which the latent class variable accounts for all observed covariance patterns in the data. That is, within individual classes, there is zero covariance and dependence among the observed indicator variables. Finally, the general idea is that each latent class corresponds to a subpopulation that has its own set of parameter values. However, the class membership of individuals in the population is not known but latent (unobserved); it is to be inferred from data using the parameters of the LCA model. Let us consider the case of survey item–response data. This analysis results in subgroups of individuals who are most like each other (e.g., individuals who belong to the same class have responded to the survey items similarly) and are most distinct from those in other classes (i.e., who present different response patterns). While a detailed explanation of the LCA model formula is beyond the scope of this review, we refer interested readers to Nylund-Gibson and Choi (2018), who provide a clear and accessible presentation of the mathematical formulation of LCA in the section “What is Latent Class Analysis (LCA)?” (pp. 441–443).

LCA differs from other latent variable mixture modeling methods because it is an analysis performed with cross-sectional data and categorical indicators that are binary, polytomous, ordered/unordered categorical, count, or a combination of these data types. While some scholars suggest that LCA should include only categorical indicators (McLachlan & Peel, 2000), we concur with others who specify that LCA can also handle continuous and categorical variables in the same model (Aflaki et al., 2023). Consequently, having *at least* one categorical variable is a distinctive characteristic of LCA (Sinha et al., 2021). Suppose data are cross-sectional and indicators are all continuous. In that case, the latent variable mixture modeling method is referred to as latent profile analysis (LPA), and the obtained groups are referred to as profiles instead of classes (Killian et al., 2019). The current study aims to systematically review the use of LCA in psychology research; hence, latent profile analyses will not be included.

## Latent class analysis as a multistep analysis

Despite LCA being considered “the simplest type of mixture model” (Whittaker & Miller, 2021, p. 376), it is a complex and multistep statistical analysis. LCA was first introduced in 1950 (Lazarsfeld, 1950) and has since undergone a number of revisions and advancements (for a complete summary, see Clogg, 1981, 1995; Hagenaars, 1990; Vermunt, 1997; Nylund-Gibson & Choi, 2018). As LCA continues to evolve, different tutorial papers have been published with the aim of offering guidelines to researchers interested in conducting such analysis (Aflaki et al., 2023; Masyn, 2013; Nylund-Gibson & Choi, 2018; Sinha et al., 2021; Weller et al., 2020). Across those tutorial papers, it is possible to identify the following steps of the LCA:

- *Preliminary methodological decisions* (e.g., Weller et al., 2020): Before conducting LCA, researchers need to make several methodological decisions. These include determining the number of cases to sample, selecting indicators to measure the phenomenon of interest, choosing software, selecting the most appropriate estimation method for their data, and deciding how to handle missing data.
- *Class enumeration process* (e.g., Nylund-Gibson & Choi, 2018): The main goal of LCA is to identify the minimum number of latent classes, represented by one or more categorical latent variables, that can explain the relationships among a set of observed variables. The decision regarding the number of classes to be used in the analysis, referred to as the class enumeration problem, involves a combination of statistical and substantive considerations and shares many concepts in common with the process of choosing the number of factors in factor analysis, not the least of which is that the class enumeration process is a critical but subjective decision in LCA. That is, the idea that there is a correct number of classes implies that there is a single, definitive number of classes that perfectly describes population diversity. However, in the practice of LCA, there is no one true model. Moreover, if there were, the true model would likely be highly complex and would only capture the data-generating process at a specific moment in time when the data were collected. Therefore, all models are considered to be inaccurate, and the best we can hope for is that a model provides an approximation of the data-generating process that is close enough to be useful (Box, 1976). With this in mind, after making the initial methodological decisions, researchers begin the process of class enumeration to determine the number of classes to be included in the LCA model for analysis. Many studies using LCA have opted to compare multiple class solutions by starting with a one-class model and incrementally adding classes until the model fits the data well using various fit statistics and diagnostic measures. This process involves fitting several latent class models with different numbers of latent classes. Ultimately, it is necessary to compare several fit indices to identify a small number, preferably three or fewer, potential best-candidate latent class models based on the model fit statistics for further consideration.

- *Closer inspection of the best LCA solutions* (e.g., Masyn, 2013): After the researchers have identified the three or fewer best LCA solutions based on fit indices, they need to assess the quality of these solutions. It is important to note that an LCA model can fit the data well but still have limited accuracy in assigning cases to categories. Researchers should carefully examine the top LCA solutions to evaluate their classification accuracy and interpretability.
- *Selection of the final LCA model* (e.g., Sinha et al., 2021): When researchers find a good-quality LCA solution, they can consider it as the final solution and should describe it thoroughly. Each class is defined by a different pattern of response to indicators through item–response probability. In simpler terms, the LCA solution shows the probability (e.g., 73%) that cases assigned to a class (e.g., class 1) have to give a specific answer (e.g., yes) to each indicator included in the analysis. It is important to note that each individual is assigned to a class based on probability; individual posterior probabilities indicate the probability that a case belongs to each class. For example, in a three-class solution, case X may have a 10% probability of belonging to class 1, 8% to class 2, and 82% to class 3. Although it could be considered primarily assigned to class 3, the latent nature of the class variable takes into account the uncertainty of class membership.
- *Optional steps:* Once the final LCA solution is identified and described, the researchers may consider their analysis concluded. At the same time, different methodological tutorials have stressed that researchers should offer other evidence about their LCA final solution, for example, by validating the same solution in a different sample (Masyn, 2013) or by verifying whether covariates differentially predicted class membership (e.g., Weller et al., 2020).

Given its relatively recent development, its continuous revisions and advancements, and its complex and multistep nature, the research practice in conducting LCA is still inconsistent across studies (Killian et al., 2019; Ulbricht et al., 2018).

## Latent class analysis in psychology

In recent decades, there has been a significant increase in the use of LCA across various scientific disciplines. In particular, notable growth has occurred in the application of mixture models, including LCA, in the behavioral and educational sciences over the past few decades (Masyn, 2013). This growth can be attributed to both the increased availability of software programs capable of running this analysis and advancements in high-speed computing (Masyn, 2013). The expansion of LCA studies also stems from the valuable opportunities it presents for scientific investigation. The classification of cases into homogeneous groups has important implications in fields such as education, medicine, psychology, and business, where identifying different types of individuals is of particular interest (Morgan, 2015).

The use of LCA has been increasing across various fields, including medicine (Sinha et al., 2021), social work (Killian et al., 2019), education, and psychology (Nylund-Gibson & Choi, 2018). While it is recognized that LCA is becoming more common, only a few studies have systematically attempted to review how this analysis is conducted and reported. To date, four systematic reviews have been published on LCA studies (Killian et al., 2019; Petersen et al., 2019; Ulbricht et al., 2018; Zhou et al., 2018). Killian et al. (2019) reviewed 32 studies, 59.4% of which used LCA, published in social work journals. Petersen et al. (2019) reviewed 23 studies that used LCA or LPA to study children's mental health. Ulbricht et al. (2018) reviewed 28 studies using LCA to explore subtypes of depression among adults experiencing depression symptoms. Lastly, Zhou et al. (2018) reviewed 99 studies, 92.9% of which used LCA, to study patient and general population healthcare preferences.

The reviews often focused on specific constructs, such as mental health and depression, that are relevant to the field of psychology. This construct focus supports Nylund-Gibson and Choi’s (2018) statement that LCA “is an analytic technique that has become increasingly popular among psychological researchers” (p. 4).

What is lacking is a comprehensive review that thoroughly explores the application of LCA in psychology, with a specific emphasis on the methodological and statistical characteristics of these studies rather than focusing solely on their results concerning the advancement of knowledge for a particular psychological construct. Such a review has the potential to provide psychology researchers with the necessary theoretical and statistical groundwork to enhance the quality of their use of LCA models.

## The current study

The purpose of this systematic review was to document the use of LCA in psychology, mapping both the prevalence and quality of LCA studies. To map their prevalence, we reviewed characteristics of LCA records in terms of publications (e.g., year, journal). To assess their quality, we reviewed how authors conducted each of the LCA steps reported above. For each step, we indicated what tutorial papers suggest doing (*Current guidelines*) and what reviewed studies actually did (*Review of psychology studies*). We then synthesized our reflections and suggestions into a final section (*Best practices and recommendations*), where we integrate best practices from previous tutorials, resolve inconsistencies, update guidance with recent developments, and propose ways to reduce the gap between recommended and common research practices.

We anticipate that this systematic review of LCA studies in psychology will be a useful tool both for psychology researchers who want to conduct LCA studies and for those who will have peer-reviewed such studies.

## Methods

This review was conducted according to the principles of the PRISMA (Preferred Reporting Items for Systematic reviews and Meta-Analyses) statement (Moher et al., 2009) and followed PRISMA guidelines for reporting systematic reviews (Page et al., 2021).

### Search strategy and inclusion criteria

We conducted a comprehensive search across multiple databases, including PsycInfo, Scopus, Psychology Database, Web of Science, and JSTOR. We excluded databases not categorized under “psychology” (e.g., *Business Source Complete*). Within each selected database, we limited the search to publications categorized in psychology as a discipline. We specifically searched for the expression “latent class analysis” in the abstracts of peer-reviewed articles. Additionally, we limited our search to records published after 2013, covering the last 10 years, to determine whether recent publications were aligned with guidelines proposed by LCA tutorials. This additional limitation is also consistent with a previous review (Killian et al., 2019), which found that the majority of studies applying the latent variable mixture modeling approach were published after 2012.

When setting the guidelines for our review, we used specific criteria to include and exclude articles. Following the approach of other reviews (Killian et al., 2019; Petersen et al., 2019; Ulbricht et al., 2018), we excluded articles written in languages other than English. In terms of methodology, we only included studies that actually conducted LCA and excluded studies that mentioned LCA in the abstract but performed a different analysis, such as LPA, or that were not empirical studies (e.g., commentary, tutorials). Finally, we excluded records for which the complete study in a full-text format was not available, for example, studies that were only presented through a conference presentation.

### Study screening and review

**Fig. 1:**
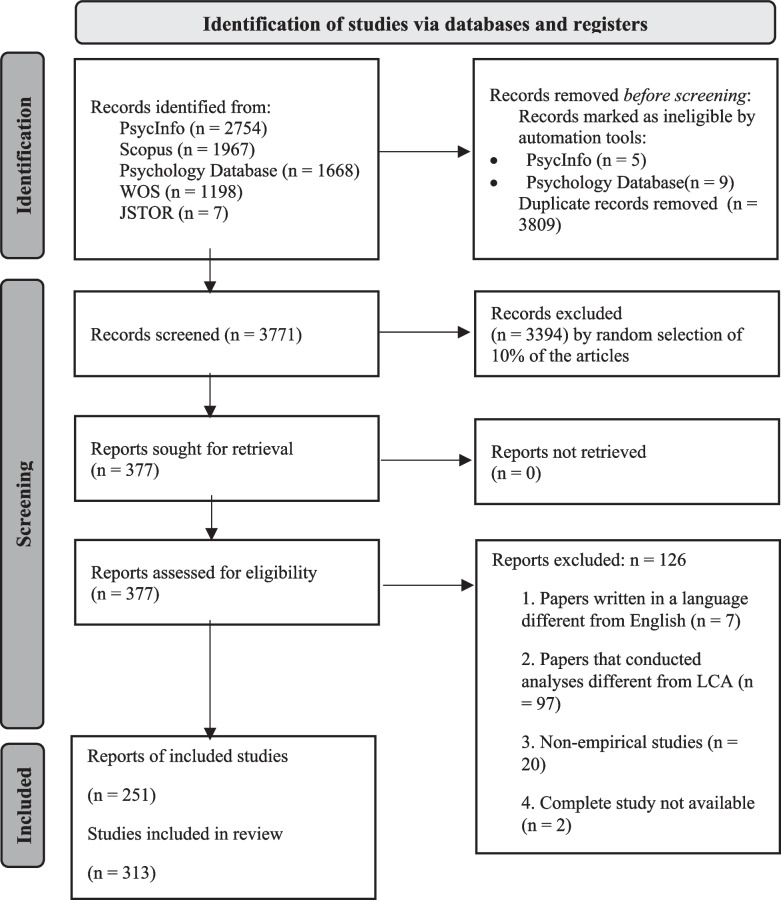
PRISMA flow diagram illustrating the flow of information at each stage of the review process

As reported in Fig. 1, our search—conducted on January 23, 2023—yielded 7,580 results across PsycInfo (*n* = 2,754), Scopus (*n* = 1,967), Psychology Database (*n* = 1,668), Web of Science (*n* = 1,198), and JSTOR (*n* = 7). Automated tools identified five articles from PsycInfo and nine from the Psychology Database as ineligible, leaving 7,580 records. After removing duplicates (*n* = 3,809), 3,771 articles were identified. The complete list can be found in the supplementary materials (File 1) available at https://osf.io/26xps/files/osfstorage.

Building on the rigorous approach of previous reviews (Egger et al., 1998), we randomly selected 10% of the articles, totaling 377 records, for eligibility assessment. This method ensures a comprehensive representation of LCA publications in psychology, significantly reducing the number of records requiring review. After downloading of these articles, both abstract and full-text readings led to the exclusion of 126 records for various reasons:

1. Seven articles were written in a language other than English (e.g., Ling et al., 2015; Lorenz et al., 2020).
2. We excluded 97 articles that did not conduct an LCA despite mentioning it in their abstracts. Specifically, 88 articles were excluded because they used a different analysis method belonging to the family of latent variable mixture modeling approaches. Among these, 58 articles (65.9%) conducted a latent profile analysis, 14 (15.9%) conducted a longitudinal latent class analysis, six (6.8%) conducted a latent transition analysis, five (5.7%) conducted a longitudinal latent profile analysis, four (4.5%) conducted a multilevel latent class analysis, and one (1.1%) conducted a growth mixture model. We primarily excluded papers where authors explicitly stated that they performed an analysis other from LCA. Additionally, there were cases where authors claimed to have conducted LCA when the analyses they carried out did not fit the LCA definition—for example, in cases where only quantitative indicators were used, and profiles were based on indicator means (latent profile analysis) rather than item–response probabilities (latent class analysis). Finally, nine articles were excluded because they did not directly perform any LCA or finite mixture model. For example, articles that performed a systematic review included different articles, some of which performed an LCA, as cited in the abstract.
3. We excluded 20 non-empirical studies, including theoretical papers (e.g., Collier & Leite, 2017), psychometric studies (e.g., Brusco et al., 2017), tutorials (e.g., Aflaki et al., 2023), research protocols (e.g., Shanahan et al., 2019), and commentaries (e.g., Jenness & McLaughlin, 2015).
4. Two records were excluded because their complete study in a full-text format was not available, for example, when the record consisted of a conference presentation (e.g., Dakin et al., 2022).

After exclusion of these articles, the total number of articles included in the review was 251. However, some studies conducted more than one LCA, resulting in a total of 313 coded LCAs. In the following pages, we will use the terms “articles/reports/publications” to refer to the 251 records included in the review and the terms “studies/analyses” to refer to each specific LCA conducted across the 251 records.

The selection process from 7,580 records to 251 articles (see Fig. 1) was carried out by two authors (AS and RC). These authors independently read all the abstracts of the 10% of studies randomly selected from the original list and evaluated the eligibility of each record (the complete list of articles that underwent the selection process and the corresponding reason for exclusion can be found in the supplementary materials [File 2] available at https://osf.io/26xps/files/osfstorage). Disagreement between reviewers was resolved through team meetings. In some cases, the entire article was reviewed to ensure an accurate evaluation of the record.

### Data extraction

We utilized a Microsoft Excel spreadsheet to create customized data extraction forms. The development of the data extraction form for the articles involved a systematic and iterative approach, which included collaborative meetings with the entire research team. AS, RC, and MR coded the first five articles, enabling thorough testing and refinement of the coding template through discussions within the entire research team. Following this, AS, RC, and MR coded an additional seven articles, and the entire team discussed further modifications to the coding template.

Once the data extraction form was finalized, 12 articles were independently coded by AS, RC, and MR. The agreement among independent coders was calculated using Cohen's kappa coefficient (Cohen, 1960), where higher values (range 0–1) suggest more robust agreement. The agreement among the three independent coders was 0.935.

Given the satisfactory level of agreement, subsequent coding was conducted individually by two authors: RC coded articles registered in “File 2” with an even number, and MR coded those with an odd number. Any uncertainties in coding were thoroughly discussed with AS. The results of the extraction process can be found in the supplementary materials (File 3) available at https://osf.io/26xps/files/osfstorage.

We extracted the following information from each record and its studies:

1. Publication details: year, journal, and database of publication;
2. Methodological decisions: sample size, representativeness, indicators, software, estimation method, and missing data management;
3. Class enumeration process: the number of LCA models run and compared and the fit indices used to compare models;
4. Evaluation of the best LCA solutions: interpretability, classification diagnostics;
5. Selection of the final LCA model: description of the selected model in terms of the number of classes, quality, and details provided by authors about the selected solutions;
6. Optional steps: validation of the LCA model, association between class membership and covariates.

Except for the first type of information (publications), all information pertains to the typical process of conducting LCA in psychology. We will outline how published LCA tutorials recommend carrying out each step and compare these recommendations to the actual practices observed in reviewed studies. Based on this comparison, we will present a final section with best practices and recommendations to guide future applications of LCA in psychology.

## Results

Results for the 251 records included in this review, along with their respective 313 LCAs, are presented separately for the six different types of information we gathered. The findings are based on the analysis conducted in SPSS, version 28 (IBM Corp, 2021), using an SPSS data file (File 4) and its syntax file (File 5), which are in the supplementary materials available at https://osf.io/26xps/files/osfstorage.

### Publications included and their characteristics

The review analyzed 251 records published between 2013 and 2023 from peer-reviewed publications. Excluding 2023, where only three records were found due to the search being conducted in January 2023, we found an average of 25 records per year (*M* = 25.0; *SD* = 9.70). In 2014, there were eight LCA publications, while in 2020 and 2022, there were 36 publications each, showing an increasing trend in the use of LCA in psychology. Similar trends were observed in social work, education, and psychology journals by Killian et al. (2019) and Nylund-Gibson and Choi (2018).

The 251 records were published across 162 different scientific journals, with 75.92% of the journals represented by just one article. The top journals publishing LCA studies are *Child Abuse & Neglect* (*n* = 9 out of 251; 3.6%), *Journal of Affective Disorders* (*n* = 8; 3.2%), *Drug and Alcohol Dependence* (*n* = 7; 2.8%), and *BMJ Open* (*n* = 7; 2.8%).

In terms of database identification, most records (59.40%) were identified by more than one database. The database that identified the most records was PsycInfo (183 out of 251 records; 72.9%), followed by Scopus (50.6%), Psychology Database (47.8%), and Web of Science (29.1%). JSTOR was unable to identify any of the 251 publications included in our review.

### Step 1: LCA methodological decisions

Conducting LCA requires researchers to make several statistical and theoretical decisions before running the analysis. These decisions often intersect; however, for clarity, we discuss them as discrete steps.

#### Sample

##### **Current guidelines**

In various sources discussing LCA, there are differing recommendations regarding the ideal sample size. Killian et al. (2019) and Weller et al. (2020) suggest a minimum of 300 cases, while Whittaker and Miller (2021) propose a sample size of over 400 to enhance the accuracy of the class identification process. On the other hand, Nylund-Gibson and Choi (2018), Sinha et al. (2021), and Aflaki et al. (2023) recommend a heuristic of at least 500 cases to ensure that commonly used fit indices for mixture models work effectively. Additionally, Masyn (2013) emphasizes the importance of sample representativeness. The identified classes and their proportions reflect the distribution of latent classes in the sample, not in the entire population. Therefore, researchers must interpret estimated class proportions cautiously when using a nonrepresentative sample, as a class with a small proportion may represent a pattern that is rare in the nonrepresentative sample but more common in the actual population.

##### **Review of psychology studies**

We identified nine studies that did not report the *size* of their sample. In the remaining 304 LCAs, sample sizes ranged from 40 (Pandya, 2023) to 3,622,424 participants (Gebregziabher et al., 2018). The median sample size was 1,234 (*M* = 24,133; *SD* = 218,668.77). Almost one-third of the studies (98 out of 304; 32.24%) used a sample size smaller than the suggested cutoff of 500 cases. In agreement with the finding from Killian et al.’s (2019) review, the sample size was significantly correlated with the number of classes in the final reported LCA models (Spearman’s ρ = .405, *p* < .001), with greater sample size related to increased unobserved heterogeneity and number of classes. The same relationship was not found in the Zhou et al. (2018) review.

Regarding *representativeness*, 1.9% of the studies (*n* = 6) adopted a sample that coincided with the population itself; for example, Fox et al. (2021) aimed to identify typologies of homicides using a registry containing *all* the cases of national homicides. Among the remaining studies, in 20.2% of cases, the authors stated that their sample was representative of the population, although only one-third of these studies (35.5%) explicitly stated why and how the sample was representative (e.g., specifying the adopted sampling procedure; Lentz et al., 2019). The representativeness of the samples was never investigated in previous reviews.

#### Indicators

##### Current guidelines

Once the sample has been defined, researchers must select the indicator variables that will be used to define the hypothesized unobserved classes. Most of the tutorials offered suggestions about the *number* of items to include in the LCA. While some scholars stressed that more indicator variables lead to better results (Aflaki et al., 2023; Weller et al., 2020), others specified that including too many indicators can be computationally demanding (Sinha et al., 2021). Simulation studies (e.g., Whittaker & Miller, 2021; Wurpts & Geiser, 2014) recommended that the number of indicators used in LCA should be greater than five when possible. Nylund-Gibson and Choi (2018) stressed that it is not just a matter of the number of indicators, but their quality should also be considered. Adding items that are highly discriminating among the classes (i.e., separate well between the classes) to an LCA has been shown to improve correct latent class recovery and to help mitigate the destabilizing effects of a small sample size (Wurpts & Geiser, 2014). If the researchers have “good” items that separate classes well, then they do not need as many items. This observation is why item choice in an LCA should be motivated by well-defined research questions and a strong theoretical background (Nylund-Gibson & Choi, 2018; Sinha et al., 2021).

Regarding the *nature* of indicators, LCA is usually adopted with categorical data. However, it is important to stress that LCA can handle continuous and categorical variables in the same model (Aflaki et al., 2023). Similarly, when only categorical data are adopted, researchers can adopt items with different scales (e.g., dichotomous and polytomous) in the same LCA (Sinha et al., 2021).

##### **Review of psychology studies**

In our analysis of 313 LCA studies, three studies did not provide any details about the indicators they used for the LCA. This lack of information was also noted in previous reviews (Ulbricht et al., 2018). In the remaining 310 LCA studies, the authors used a range of indicators from 3 to 63 (*Mdn* = 9.5, *M* = 11.86; *SD* = 7.98). Most studies (276 out of 310; 89.03%) used more than five indicators.

In our review, we found no significant correlation between the number of indicators used in the analyses and the number of classes in the final LCA solution (Spearman’s ρ = .093, *p* = .103), which supports the findings from Killian et al. (2019)’s review.

Regarding the nature of the indicators, most LCAs (86.5%) used indicators that were all measured on the same scale. Specifically, the majority of these studies used only dichotomous indicators (88.3%), as also found in a previous review (Ulbricht et al., 2018). Few studies used only unordered (2.6%) or only ordered (10.9%) categorical indicators. We also found that 13.5% of the LCAs were based on a mix of indicators with different natures, including dichotomous (83.3%), unordered categorical (19.5%), ordered categorical (71.4%), and continuous (27.5%) indicators.

#### Software

##### **Current guidelines**

As reported in the different LCA tutorial papers (e.g., Masyn, 2013; Nylund-Gibson & Choi, 2018; Sinha et al., 2021; Weller et al., 2020), LCA can be conducted using several commercial and free statistical software programs. The software packages cited across the tutorial papers were as follows: STATA (StataCorp, 2023), SAS (SAS Institute Inc., 2020), R (R Core Team, 2023), Mplus (L. K. Muthén & Muthén, 1998–2017), and LatentGOLD (Vermunt & Magidson, 2016).

##### **Review of psychology studies**

Among the 251 papers we reviewed, 15 did not report the software they adopted to conduct the LCA. Among the remaining papers, almost two-thirds used Mplus software (63.1%). Other commonly used software programs were LatentGOLD (10.2%), SAS (11.9%), R (8.1%), and Stata (5.1%). We also found two studies using Sawtooth Software (Jürkenbeck et al., 2019; Van Cauwenberg et al., 2016) and one using WinMira and LEM (Rivera-Navarro et al., 2018). Finally, we reviewed one paper (Hancock et al., 2021) in which the same LCA models were run using both SAS and Mplus.

#### Estimation method

##### Current guidelines

Statistical methods can be performed using different estimators. These estimators are usually classified as reflecting a classical frequentist paradigm, such as maximum likelihood estimators or Bayesian methods. While Bayesian statistics have become more popular, researchers in the field of psychology mostly use frequentist statistics (van de Schoot et al., 2017). In the use of LCA, these non-Bayesian statistical tools often include variants of weighted least squares and those based on likelihood theory, with the latter being the most dominant.

Software programs typically have a default estimator (Weller et al., 2020). Therefore, not surprisingly, given our earlier observation, the maximum likelihood (ML) estimator, obtained via the expectation–maximization (EM) procedure, is the default for the LCA model parameters in most software (Nylund-Gibson & Choi, 2018). This estimator also has a robust variant that provides ML estimates with standard errors and a chi-square test statistic (when applicable) that is robust to nonnormality. This is the default estimator (labeled MLR) for LCA in the widely used Mplus software (L. K. Muthén & Muthén, 1998–2017).

Before performing LCA in the chosen software, researchers must determine which estimator to use. It is well known in the statistical research literature that the desirable properties of the estimators depend on the combination of common conditions, as follows:

1. Whether the purpose of the LCA is to capture and analyze complex relationships, account for measurement errors, make predictions or inferences about the latent variables, or some combination of these purposes.
2. The nature and distribution of the indicators, such as continuous ordered categorical, binary, unordered multinomial, censored, or count.
3. The sample size, although researchers face two main challenges when selecting an estimator based solely on the minimum sample size required to fit an LCA adequately. Firstly, establishing a minimum adequate sample size for an LCA involves considering multiple nuanced factors, over and above the choice of estimator, that affect the estimator’s performance in LCA. Secondly, recent research (McNeish, 2016) has shown that Bayesian methods are being favored over frequentist methods for handling smaller sample sizes more effectively. However, while Bayesian methods are better equipped to handle small sample situations, simply switching to a Bayesian framework alone does not necessarily resolve the small sample problem and may result in more biased estimates than frequentist methods, as demonstrated by McNeish.
4. The method that will be used to handle their missing data, which, of course, may depend on the missing data mechanism (see next section).
5. The data structure, such as nested or multilevel data, such as the one typically found in educational settings (students in classrooms).
6. The number of parameters to be estimated, which is related to the complexity or size of the LCA model.
7. Whether or not the analyst prefers Bayesian methods for foundational statistical or philosophical reasons; for example, Bayesian modeling is described as a coherent framework for inference (Gelman et al., 2013).

As an example, we imagine an LCA of ordered categorical observed variables that arise from responses to the items of a psychological instrument, with each item having a five-point item–response scale. In this case, Bayesian estimates are generally closer to the true values. They are less variable than maximum likelihood estimates, particularly for a small number of items, a small number of respondents to the psychological instrument, or both. With larger numbers for either, Bayesian estimates and ML estimates produce similar results because the posterior is dominated by the likelihood (Rupp et al., 2004). Additionally, the weighted least-squares estimator can be used for censored or categorical data (Killian et al., 2019), or Bayesian estimation methods can be used when model assumptions such as local independence are not met (Nylund-Gibson & Choi, 2018).

##### Numerical convergence and multiple starts to the algorithm

An interesting feature of applications of maximum likelihood theory is that the maximization problem rarely has an analytical solution wherein it is possible to write the maximum likelihood estimator explicitly as a function of the data (for more details, see Masyn, 2013). Rather than an analytical solution, the typical uses of ML or MLR estimators implement numerical algorithms for the maximization of the log-likelihood that involves an iterative approach resulting in successive sets of parameter estimates.

The properties of the log-likelihood function and the algorithm used often ensure that the proposed solution will converge to the true solution. Ensuring that the proposed maximum likelihood solution will converge means that the difference between the two solutions can be made very small by running a large number of iterations. However, it is important to note that this numerical convergence is only sometimes guaranteed on a theoretical basis due to difficulties in studying the properties of the log-likelihood function or insufficient evidence to prove numerical convergence given the chosen algorithm.

When it is not possible to ensure numerical convergence theoretically, a heuristic approach is usually followed. That is, a numerical optimization algorithm is run several times with different, and possibly random, starting values for the parameters. The reasoning is that if all the runs of the algorithm (or a majority of them) lead to the same proposed solution (up to small numerical differences), then this can be taken as evidence that the proposed solution is a good approximation of the true solution. This iterative process can be set by the researchers, including the so-called random starting values (Masyn, 2013). The use of a set of random start values allows researchers to estimate the model many times to verify whether, across the set of random start values, there is convergence on a similar solution (Nylund-Gibson & Choi, 2018). This estimator should solve the problem of model estimates arrived at as a product of local maxima (Killian et al., 2019). Local maxima occur when parameter estimates in a model converge to arrive at a possible answer but not the best overall or global estimate (Lanza et al., 2003). Even though most of the guidelines raise the issue of starting values, few offer specific recommendations about how to set the random starting values. The only exceptions are Masyn (2013), who suggested using 50–100, or higher, starting values, and Morgan (2015), who stated that in some of the more complex LCA models, the number of random starting values and optimization steps should be increased to as many as 2,000 and 500, respectively.

##### **Review of psychology studies**

Results of our review confirm what was found in previous reviews (i.e., Ulbricht et al., 2018; p. 238): “the estimation method and starting values used to conduct LCA were missing more often than not.” In particular, we found that 77.3% of the studies did not report the method used for estimation. For those who reported it (*n* = 71), it was often ML (47.89%) or MLR (45.07%). In five cases (7.04%), authors reported using the sandwich estimator.

Similarly, starting values were not reported in 84% of the analysis. As both estimators and starting values (as well as missing method, see next section) can be inferred from the syntax files, we also checked how many papers did report the code of their analysis (e.g., as supplementary or open materials); unfortunately, only five studies out of 313 (1.6%) provided this material.

#### Missing data

##### Current Guidelines

According to Sinha et al. (2021), three methods are widely used to deal with missing variables when performing LCA: deletion, multiple imputation, and full information maximum likelihood (FIML). The first method includes only complete cases in the analysis, but this implies that large swathes of data can be lost and that a large portion of participants is not represented anymore in the final sample. In general, this is the least preferred of the three methods and should seldom be used (Sinha et al., 2021). In relation to LCA, the other two methods have their inherent advantages and disadvantages. Both approaches work on the assumption that the data are either missing completely at random (MCAR) or missing at random (MAR) (Masyn, 2013). The ML (and MLR) estimators automatically adopt the FIML method to manage missing data, i.e., individual cases are not excluded from the analysis unless they are missing data on all the observed items. Aflaki et al. (2023) also report a solution different from the three reported above (i.e., deletion, multiple imputation, and FIML). They suggested treating “variables containing missing values as categorical variables, where missing values are treated as bring in their own category” (p. 171).

##### Review of Psychology Studies

Results of our review indicate that almost all the studies (82.7%) did not report information about the method they adopted to manage missing data. Among the 54 studies that reported information about the method they adopted to manage missing data, most (81.48%) adopted the FIML, which is in agreement with the finding from Killian et al. (2019). Other studies reported handling missing data within the expectation-maximization algorithm (0.55%) and listwise deletion (0.74%). Only two studies reported the use of multiple imputation (Bayly et al., 2022; Yuan et al., 2022), and one study reported the use of the Maximum Likelihood with Missing Values (MLMV; Harrison et al., 2022) method.

### Step 2: Class enumeration

Once the researchers have made the decision reported above, they are ready to run different LCA models in order to determine the number of classes that best represent the heterogeneity of their sample. Deciding on the number of classes is often the most arduous phase of the mixture modeling process (Masyn, 2013). It is labor-intensive because it requires the estimation of a set of models with a varying number of classes. It is complicated in that the selection of the “best” model(s) from the set of models under consideration requires the examination of a host of fit indices (Masyn, 2013). In sum, at this stage, the researchers have to decide how many LCA models they want to run as well as the criteria they want to use to evaluate and compare those models.

#### How many LCA models should be run and compared?

##### Current guidelines

Tutorial papers (Masyn, 2013; Sinha et al., 2021; Weller et al., 2020) agree that researchers should begin by specifying a one-class LCA model and then fitting additional models, incrementing the number of classes by 1. Less agreement is found about when the researchers should stop testing additional models. Some scholars suggest proceeding until the models are no longer well identified (e.g., a local instead of a global solution is found; Masyn, 2013), while others suggest proceeding until the best model is identified, that is, when the researcher observes that after a well-fitting model, the quality begins to deteriorate (Weller et al., 2020). Others (i.e., Aflaki et al., 2023; Sinha et al., 2021) instead do not provide any specific stopping rule, as the “correct number of models to fit the data will largely be dictated by the sample size, number and quality of indicators used in the model, and what an acceptable size may be for the smallest class” (Sinha et al., 2021; p. 8).

##### **Review of psychology studies**

Across the 313 LCAs we reviewed, 34 (10.9%) did not report how many LCA models were run and compared. Among the remaining 279 LCAs, researchers performed class enumeration running from 1 to 15 LCA models (*Mdn* = 5; *M* = 5.48; *SD* = 1.94). Across those studies, only 60.2% included the one-class solution among the solutions that were run and compared. Furthermore, only 11.0% of the studies specified which rule was adopted to decide when to stop running additional LCA models. For example, some stopped when the models were no longer well identified (e.g., “the best loglikelihood was not replicated”; Ross et al., 2018), others when the model fit began to deteriorate (e.g., “the BIC [Bayesian information criterion] started to turn bigger and/or LMRT and BLRT turned nonsignificant”; Xi, 2016), or when LCA solutions included classes that were too small (“the smallest latent group consisted of less than 1% of the sample”; Lee & Ryff, 2019).

#### Fit indices to compare LCA models

##### Current guidelines

After running different LCA models, researchers need to select the best LCA solution(s). As in conventional structural equation modeling, there is no single fit index that is agreed upon for use in enumerating classes; instead, researchers use a set of fit indices to make decisions. Masyn (2013) differentiated among three groups of fit indices: absolute fit indices, inferential relative fit indices, and information-heuristic comparisons of relative fit.

*Absolute fit indices.* Absolute fit indices compare the model’s representation of the data to the actual data. Thus, when evaluating the absolute fit of an LCA model, the model-estimated frequencies are compared to the observed frequencies across all the response patterns.

The most common test of absolute fit of the LCA model is the likelihood ratio chi-square goodness-of-fit test (Agresti, 2002; Collins & Lanza, 2010; McCutcheon, 1987). This test statistic, denoted as ${\chi }_{LR}^{2}$*, *${\chi }_{LRT}^{2}$*, G*^2^, or *L*^2^, can be interpreted as follows: failure to reject the null hypothesis implies adequate model data consistency; rejection of the null implies that the model does not adequately fit the data. Another way to inspect the closeness of fit for LCA models is by examining the standardized residuals, because the LCA residuals are constructed using the same information that goes into the overall goodness-of-fit test statistic: the model-estimated response pattern frequencies and the observed frequencies (Masyn, 2013). In other words, standardized residuals with large values (e.g., > 3) indicated response patterns that were more poorly fit, contributing the most to the ${\chi }_{LR}^{2}.$ According to Masyn (2013), having more than 5% of standardized residuals that exceed the cutoff (3) can be considered an indication of a poor-fitting model. In sum, for absolute fit, the “best” model should be the model with the fewest number of classes that has an adequate overall goodness of fit (i.e., nonsignificant ${\chi }_{LR}^{2}$ test and less than 5% of standardized residuals with large values).

Morgan (2015) and Schreiber (2017) suggest evaluating the absolute fit and also checking the log-likelihood value. Finally, Schreiber (2017) suggests also considering the number of parameters in the model (Npar), as it can be part of the evaluation for all models where the likelihood ratio chi-square is not significant (*p* > 0.05).

*Inferential relative fit indices*. The expression “relative fit indices” is used to refer to indices that compare the model’s representation of the data to another model’s representation. These indices are referred to as “inferential” when they are associated with a likelihood-based test that compares just two neighboring class models (e.g., a two-class versus a three-class model) at a time and indicates whether these two models are significantly different (*p* < .05), i.e., whether adding a class leads to a statistically significant improvement in model fit. If they are not significantly different (*p* ≥ .05), the model with fewer classes should be preferred. Three tests belong to this group of fit indices: the Vuong–Lo–Mendell–Rubin likelihood ratio test (VLMR; Lo et al., 2001), an adjusted VLMR test (aVLMR: Lo et al., 2001), and the bootstrapped likelihood ratio test (BLRT; McLachlan & Peel, 2000). Whereas the two VLMR tests use an approximation to test for model fit, the BLRT uses parametric bootstrap methods to estimate the distribution (Killian et al., 2019). The classic VLMR test is rarely suggested across the tutorial papers, as its adjusted version is preferred because it was designed to reduce the potential of inflated type I error rates of the classic one, which was found in particular with small sample sizes (Whittaker & Miller, 2021). It must be noted that both the classic and the adjusted VLMR rely on multivariate normality of the outcomes and covariates in the models. It will favor models with a higher number of classes when these assumptions are violated (Whittaker & Miller, 2021). Furthermore, Morgan (2015) reported that these two tests performed slightly better when the indicators consisted of more continuous variables (Morgan, 2015). Similarly, in a Monte Carlo simulation of LCA using only categorical indicators, Nylund and colleagues (2007) found that the BLRT consistently outperformed the simple VLMR. According to Aflaki et al. (2023), the BLRT is not advised for models with mixed indicator data types.

*Information-heuristic comparisons of relative fit.* While the inferential relative model fit tests allow for the comparison of only two models at a time, the information-heuristic tools (also known as information criteria, ICs) enable the comparison of relative fit across a set of models. These indices of relative fit are based on *information criteria* that weigh the fit of the model (as captured by the maximum log-likelihood value) in consideration of the model complexity. These indexes indeed differ in how they operationalize model complexity (see Masyn, 2013, and Nylund-Gibson & Choi, 2018, for more information). However, they can be interpreted in the same way, such that lower values indicate a better model fit. That is, the model to prefer is the one with the lowest values of ICs among the set being considered. It can be useful to plot these values for easier inspection of their trend (see, for example, Fig. 2 in Nylund-Gibson & Choi, 2018). In practice, it is not uncommon that the information-heuristic tools continue to decrease for each additional class added. In these instances, these plots can be particularly useful for inspecting for an “elbow” or point of “diminishing returns” in model fit (e.g., small decreases in the IC for each additional latent class).

The most common information criteria used for a mixture model are the Bayesian information criterion (BIC; Schwarz, 1978), for which a *sample size-adjusted* version is available (SABIC; Sclove, 1987), and the Akaike information criterion (AIC; Akaike, 1977), for which a *consistent* version is also available (CAIC; Bozdogan, 1987). The BIC performs very well in conditions when the sample size is larger (Morgan, 2015), while it tends to underestimate the number of classes if the sample size is small (< 300; Morgan, 2015; Tofighi & Enders, 2007). Consequently, the SABIC was proposed to reduce the sample size penalty presented by the BIC. The AIC tends to select overparameterized models (i.e., that overestimate the correct number of classes). The CAIC was proposed as an extension that makes AIC asymptotically consistent and penalizes overparameterization (Bozdogan, 1987).

Different simulation studies (e.g., Morgan, 2015; Nylund et al., 2007; Whittaker & Miller, 2021) have compared the accuracy of these ICs under various conditions (e.g., sample size, indicator nature, and class prevalence rates). Regarding sample size, BIC tends to perform better than AIC, especially when the sample size is large. Conversely, BIC tends to perform poorly when the sample size is small (< 300) and may be outperformed by the AIC. In these circumstances, it is advisable to present both the AIC and BIC (Nylund et al., 2007). Regarding indicators, AIC’s performance is more stable in models with more categorical indicators, BIC is more accurate in models with more continuous indicators, and SABIC performs about the same regardless of the number of categorical versus continuous indicators (Morgan, 2015). Finally, AIC was found to perform best when one of the underlying classes had a high prevalence rate, while BIC had the most difficulty identifying classes in this condition (Morgan, 2015). In sum, although most of the tutorials (Aflaki et al., 2023; Killian et al., 2019; Weller et al., 2020) agree that the BIC and the SABIC tend to perform the best among the information criteria, it is also important to take into account simulation studies (Morgan, 2015; Nylund et al., 2007; Whittaker & Miller, 2021) that clarify in which condition each IC works better.

Other than BIC, SABIC, AIC, and CAIC, the approximate weight of evidence criterion (AWE; Banfield & Raftery, 1993) is an IC that can be used to compare LCA models, but it was named only in three tutorials (Masyn, 2013; Nylund-Gibson & Choi, 2018; Weller et al., 2020) and was not included in simulation studies that compare the performance of different ICs (e.g., Morgan, 2015; Whittaker & Miller, 2021). Furthermore, Masyn (2013), Nylund-Gibson and Choi (2018), and Weller (2020) were the only papers to suggest the adoption of the approximate Bayes factor (BF) and the approximate correct model probability (cmP) when comparing LCA models. They are not strictly information criteria (ICs), but their formulas (see Masyn, 2013) include the Schwarz information criterion (SIC; Schwarz, 1978), which corresponds to −0.5 times the BIC. While the other ICs are ordinal, the BF and the cmP provide researchers with a sense of “how much” better one model is relative to another model or relative to a whole set of models (Masyn, 2013).

Specifically, the BF is a pairwise comparison of relative fit between two models, model A and model B, and the “best” model is the model with the smallest number of classes for which BF > 3 (Masyn, 2013). In contrast, the cmP allows for relative comparisons of each model to the entire set of models under consideration. The “best” model is the model with the highest value (i.e., the probability of being correct), but a model with cmP > .10 could be considered a candidate model.

##### **Review of psychology studies**

Findings from our review suggest that researchers do not utilize all the available fit indices. On average, each LCA study reports and relies on just three indices (*M* = 2.83; *SD* = 1.71; range = 0–10; *Mdn* = 3).

*Absolute fit indices.* The absolute fit indices are less considered in research practice in psychology. Only 52 LCAs (16.6%) reported at least one element (value, *df*, *p*-value) of the ${\chi }_{LR}^{2}$ . In particular, 44 studies out of 313 (14.1%) reported the values of the test, 27 studies (8.6%) reported its degrees of freedom, and 17 (5.4%) reported its *p*-value. Only eight studies out of 313 (2.6%) reported all the elements as they should be. Even fewer scholars used standardized residuals, as only one article (Lacey et al., 2015) adopted them to evaluate absolute fit indices. Finally, 114 studies (36.4%) reported the log-likelihood value for each of the tested LCA models, and 48 studies (15.3%) reported the number of free parameters present in each model.

In reviewing our LCAs, we also found seven studies (2.2%) that reported the Pearson χ^2^. Although some software programs report that this absolute model fits the LCA model (e.g., Mplus; Asparouhov & Muthén, 2008), none of the tutorial papers have named it.

*Inferential relative fit indices.* As expected, the VLMR test was rarely reported (in only 14.1% of the studies) in favor of its adjusted version, which was considered in 43.9% of the LCAs. Finally, the BLRT was reported in 24% of the studies. Its less widespread use may reflect the fact that many software packages have not incorporated it because of its computationally intensive calculations (Killian et al., 2019). Mplus computes BLRT by requesting Tech 14 in the input script (Nylund-Gibson & Choi, 2018). We indeed found that studies conducted using Mplus were significantly more likely to report the BLRT than studies conducted with other software programs (χ^2^ (1) = 9.51, *p* = .002; Cramer’s *V* = .201).

A common issue observed across studies that relied on inferential fit indices is the lack of complete reporting of the relevant information required for interpreting these tests. For the classic VLMR test, all the studies that adopted this index (14.1%) reported at least its *p*-value. Only 4.8% also reported its value (i.e., the log-likelihood difference between the two compared models), and only one study (0.3%) reported the difference across the two compared models in terms of the number of parameters. For the adjusted VLMR test, almost all the studies that adopted this index (*n* = 106) reported its *p*-values (*n* = 103; 32.9% of the 313 LCAs). In 53 LCAs (16.9%), the adjusted VLMR test value (i.e., log-likelihood difference between the two compared models) was reported together with its *p*-value; in three cases, the *p*-value was not reported (e.g., Fu & Vaughn, 2016). Similarly, for the BLRT, among the studies that adopted this index (*n* = 75), almost all reported its *p*-value (*n* = 74; 23.6% of the 313 LCAs). In 20 LCAs (6.4%), its value was also reported together with the *p*-value; for Meyers-Pantele et al. (2021), the BLRT value was reported without its *p*-value.

*Information-heuristic comparisons of relative fit.* On average, our 313 LCA studies reported only two ICs (*M* = 1.89; *SD* = 1.16; *Mdn* = 2; range = 0–5). In agreement with previous reviews (Killian et al., 2019; Petersen et al., 2019; Ulbricht et al., 2018; Zhou et al., 2018), the most commonly adopted IC was the BIC (77.0% of the studies), followed by the AIC (51.1%) and the SABIC (47.3%). Only 31 studies (9.9%) reported the CAIC, and four studies (1.3%) reported the AWE. We also found six studies (1.9%) that relied on the AIC3 (i.e., AIC that uses 3p instead of 2p as the penalty term, where p represents the number of free parameters). Despite the fact that the graphical representation of IC values (i.e., elbow plot) was suggested in many tutorials (Aflaki et al., 2023; Masyn, 2013; Nylund-Gibson & Choi, 2018; Sinha et al., 2021; Weller et al. 2020), it was rarely adopted (only 14 studies; 4.5%) in the studies we reviewed. This may be explained, in part, by the fact that this plot is mainly needed when ICs continue to decrease with the increase in classes.

Finally, we found only four (1.3%) studies (e.g., Denson & Ing, 2014; Tsaousis et al., 2020) that also adopted the approximate BF and the approximate cmP to compare LCA models.

### Step 3: Closer inspection of the best solution(s)

While fit statistics may guide model selection, even the use of several fit indices cannot provide a definitive indication of the best model. Indeed, they may contradict each other (Petersen et al., 2019). Furthermore, “it is almost always the case in practice that there will be more than one ‘best’ model identified across the different indices. Typically, the candidate models are adjacent to each other with respect to the number of classes (e.g., three-class and four-class candidate models)” (Masyn, 2013, p. 571). Furthermore, even when fit indices suggest that just one model is plausible, researchers must still evaluate whether this solution has sufficient quality, because “it is possible for a mixture model to have a good fit to the data but still have poor latent class assignment accuracy” (Masyn, 2013, p. 569). Across the tutorials, different ways have been reported to closely investigate these potential 1-3 LCA “best” solutions. These methods can be divided into two macro-groups: interpretability and classification-diagnostics.

#### Interpretability

##### **Current guidelines**

The concept of interpretability was cited in almost all the tutorials we found (Aflaki et al., 2023; Killian et al., 2019; Masyn, 2013; Nylund-Gibson & Choi, 2018; Petersen et al., 2019; Sinha et al., 2021; Weller et al., 2020). Scholars suggest that researchers should describe the classes (i.e., see which response pattern characterizes each class) of the most plausible solution(s) and *interpret* the substantive meaning of the classes in order to consider whether such classes are in line with what would be expected from theory, whether they are easy to interpret and label, or both. To graphically represent the different classes of an LCA, an item probability plot (see, for example, Fig. 3 in Nylund-Gibson & Choi, 2018) is a good way to inspect the classes visually and to examine the qualitative differences among the classes (Nylund-Gibson & Choi, 2018).

Across the different tutorials, the interpretation procedure was accompanied by other evaluations that the researchers should conduct: evaluation of class size, parsimony, and the salsa effect (e.g., forcing multiple class solutions for a population that may not have latent classes, as described in more detail below).

*Regarding class size*, most of the tutorials are specified to avoid solutions that include one or more classes that are too small. They specifically stated that each class should contain no fewer than 50 cases (e.g., Killian et al., 2019), no less than 5% of the sample, or both (e.g., Nylund-Gibson & Choi, 2018). Scholars offer different reasons to avoid small classes. For example, Sinha et al. (2021) state that a model with a small class is often a model with too many classes, because “a small class may be the result of some sort of ‘quirk’ in the data” (p. 9), such as extreme values of a single variable. In these cases, Aflaki et al. (2023) suggest transforming or scaling such variables. Nylund-Gibson and Choi (2018) instead suggest avoiding small classes because small or “rare” classes are generally difficult to recover at small sample sizes, particularly when class prevalence is highly unequal (e.g., the classes are not of equal size). Therefore, they suggested avoiding the selection of an over-extracted and potentially unstable class solution, especially when lacking a large enough sample size that may otherwise support the reliable detection of classes with low prevalence. In contrast, Weller et al. (2020) recognize that some LCA studies have selected LCA solutions that included class sizes smaller than 5% or 50 cases and conclude that there are no existing guidelines on determining class size.

The decision to avoid solutions with classes that are too small is strongly related to the concept of *parsimony*, which has been cited in different guidelines (Killian et al., 2019; Morgan, 2015; Petersen et al., 2019). Collins and Lanza (2010) described parsimony as a philosophical principle that prefers simpler models over more complex models (i.e., LCA models, which require the estimation of fewer parameters, should be preferred). In other words, if we have two LCA solutions with similar fit, but one of the two has one class more than the other and this class is small, the solution with one fewer class should be preferred. Similarly, if estimating a model with an additional class results in one class from a prior model being split into two classes in the subsequent model, researchers should weigh the model fit statistics against interpretability and practical considerations (Killian et al., 2019). Masyn (2013) likewise suggests weighing the simplicity and clarity of each candidate model and evaluating the utility of the additional classes for the less parsimonious models.

Finally, Sinha et al. (2021) and Aflaki et al. (2023) also suggest checking for the *salsa effect* when interpreting the most plausible LCA solution(s). In particular, researchers should inspect the classes to ensure that they are not merely reflective of scaled groupings driven by a single dominant indicator or by a uniform pattern across multiple indicators. Suppose a given dataset has only one class (in other words, there are no underlying mixtures of distributions). In that case, there may be some indication that a *k*-class model fits the data. However, when the profiles are plotted, they roughly form a set of parallel lines (see Fig. 5 in Sinha et al., 2021). This finding probably suggests that the dataset has been coerced into “*k* levels,” where *k* is the number of classes the algorithm attempted to fit. This phenomenon is known as the “salsa effect.” It represents scales of severity of the indicators (e.g., mild, medium, and hot levels of salsa) rather than distinct groups.

##### **Review of psychology studies**

Across the 313 LCAs we reviewed, only 45.4% (*n* = 142) referred to the concept of interpretability when selecting the best solution. Furthermore, only 10 of these studies also explicitly stated what they meant by interpretability and how they tested it (e.g., “a key factor in choosing one model over another is that the resulting class structure is substantively meaningful; in other words, the class structure is interpretable, makes logical sense, and comports with real-world behaviors”; Shigeto et al., 2021, p. 284).

We also found that none of the studies we reviewed named the salsa effect. We further verified whether researchers considered the class size when evaluating the LCA solutions. Only 13.1% of the studies (*n* = 41) stated that they took into consideration the class size in selecting the best LCA solution. Among these studies, only 24 studies explicitly reported the cutoff they used to evaluate class size. Most of the studies (21 out of 24) stated that LCA solutions with one or more classes that included less than 5% of the cases should be excluded. Three studies instead used the 1% cutoff (e.g., Fox et al., 2021; Lee & Ryff, 2019). Finally, in one study, researchers checked the class size of the LCA solutions in order to ensure they were large enough to detect a moderate effect in subsequent analysis (“power necessary”; Bayly et al., 2022, p. 202).

Across the 313 studies we reviewed, 54 (17.25%) had at least one class of the final LCA solution with a size smaller than 5% (ranging from 0.09% to 4.9%). This finding confirms Weller et al.'s (2020) statement that researchers still select the LCA models that include class sizes smaller than 5%.

#### Classification diagnostics

##### **Current guidelines**

While the evaluation of 1–3 solutions based on their interpretability may seem “subjective” (Aflaki et al., 2023, p. 172), the evaluation based on classification diagnostics is characterized by the mathematical calculations of different indexes. They are all based on estimated posterior class probabilities (i.e., the probability for each case of belonging to each class of the LCA solution)*,* so they inform in different ways about how accurate the assignment of each case to a class is. An extensive presentation of these diagnostics was provided by Masyn (2013), while other tutorials presented only a few such diagnostics. Furthermore, Masyn (2013) clearly stated that these model classification diagnostics “should *not* be used to evaluate the model-data consistency in either absolute or relative terms” (p. 569). In other words, such classification diagnostics should not be used for the class enumeration step (i.e., comparison of different models) but to closely inspect 1–3 solutions and evaluate how well these models are able to classify individuals into latent classes.

The most famous of these classification quality indexes is the relative entropy, often referred to simply as *entropy* (E). It is an index that summarizes the overall classification precision for the whole sample across all the latent classes (Ramasway et al., 1993) and ranges from 0 (when posterior classification is no better than random guessing) to 1 (when there is perfect posterior classification for all individuals in the sample). Almost all the tutorials (Aflaki et al., 2023; Killian et al., 2019; Nylund-Gibson & Choi, 2018) noted that values above .80 were acceptable. Weller et al. (2020) and Morgan (2015), on the other hand, confirmed the lack of agreement regarding the cutoff criterion for entropy (B. O. Muthén, 2008); Weller et al. (2020) also noted that it may be difficult to publish a study with an entropy value below .60. LatentGOLD software produces two indexes that summarize the overall classification accuracy of the LCA solution: entropy R-squared (which corresponds to entropy) and standard R-squared (which can be interpreted as a reliability measure; Vermunt & Magidson, 2016).

While most of the tutorials (Aflaki et al., 2023; Nylund-Gibson & Choi, 2018; Sinha et al., 2021; Weller et al., 2020) reaffirmed that entropy should not be used for class enumeration, there are still guidelines (i.e., Killian et al., 2019; Zhou et al., 2018) that erroneously present entropy together with fit indices as a means to compare LCA models and select the “best” one. Furthermore, two recent simulation studies (Morgan, 2015; Whittaker & Miller, 2021) have tested the accuracy of entropy in selecting the best LCA solutions. They confirmed that entropy should not be used for class enumeration (“Entropy exhibited poor performance in terms of class enumeration, never selecting the correct number of latent classes with 80% accuracy in any manipulated condition”; Whittaker & Miller, 2021, p. 387). Furthermore, they found that entropy values were slightly higher with smaller sample sizes, more categorical variables, and less balanced class prevalence (Morgan, 2015).

In contrast to entropy, which provides an overall summary of latent class assignment error, the following indices provide class-specific measures of classification quality. In particular, the average posterior class probability (AvePP) enables evaluation of the specific classification uncertainty for each of the latent classes, measuring how well a given model classifies individuals into their most likely class. Specifically, the AvePP corresponds to the average posterior probabilities of all participants modally assigned to a specific class based on the highest posterior class probability for the individual participant (e.g., a participant with a 60% probability of belonging to class 1, a 30% probability of belonging to class 2, and a 10% probability of belonging to class 3 will be modally assigned to class 1; Sorgente et al., 2019). AvePP is bounded by 0 and 1, and different cutoffs can be found in the tutorials. Masyn (2013) and Nylund-Gibson and Choi (2018) suggest that values above 0.70 are needed to consider the classes well separated and the latent class assignment accuracy adequate, while Weller et al. (2020) refer to researchers who used values of .80 (Weden & Zabin, 2005) and .90 (B. O. Muthén & Muthén, 2000) as a cutoff for acceptable solutions.

Another way to evaluate the classification quality consists of comparing the model-estimated proportion for class *k* (i.e., the proportion of participants that should be in the class *k* according to the estimated model), called class proportion (π_k_), with the modal class assignment proportion (mcaP_k_, that is, the proportion of participants actually assigned to that class *k*). Classification results can be considered good when the mcaP_k_ for each class is included in the 95% CI of the π_k_ (Sorgente et al., 2019). Larger discrepancies between *mcaP*_k_ and *π*_k_ are indicative of larger latent class assignment errors. This discrepancy is consistent with the “classification error” index presented by Schreiber (2017), who defined it as a means to estimate the proportion of misclassified cases.

Another class-specific measure is the odds of correct classification ratio (OCC), which weights the AvePP for the model-estimated class proportion (π) of each class. As AvePP approaches the ideal value of 1, the OCC increases. Thus, large OCC values (i.e., > 5; Nagin, 2005) for all classes indicates a latent class model with good latent class separation and high assignment accuracy.

Finally, Masyn (2013) and Nylund-Gibson and Choi (2018) specify that the classification accuracy can also be evaluated at the item level. In other words, researchers could estimate how much each LCA indicator contributes to generating classes that have high *class homogeneity* (i.e., people in the same class respond in the same way to that indicator) and *class separation* (i.e., the item can be used to distinguish between members across at least one pair of classes of the LCA solution). A class with item–response probabilities > .70 and < .30 has high homogeneity (Nylund-Gibson & Choi, 2018). Class separation with respect to a particular item can instead be calculated using the estimated item endorsement odds ratio (see Masyn, 2013, for the formula): Values between two classes of > 5 and < .20 indicate high separation. Notably, items may differentiate some classes well but not others. Researchers should thus consider holistically how well each item contributes to the overall model's class separation.

##### **Review of psychology studies**

Entropy was adopted in more than half of the studies (62.0%, *n* = 195). However, in half of these cases (46.9%), it was used incorrectly for the class enumeration procedure. Only 19.49% of the studies adopting entropy (i.e., 12.14% of the 313 reviewed studies) used it properly, i.e., to evaluate the classification accuracy of the best LCA solution(s). Finally, in 32.81% of the remaining studies, researchers did not specify the purpose (class enumeration vs. classification accuracy) for which it was adopted. This finding aligns with other studies (e.g., Tein et al., 2013), which have also found that entropy is frequently used in the class enumeration process.

The standard R-squared was adopted in just one study (Pandya, 2023) and was correctly used to evaluate classification accuracy. Forty-six studies (14.7%) reported the average posterior probability (AvePP). However, it was appropriately considered a classification diagnostic in only 17 of these studies (36.95%). In the remaining LCA studies reporting the AvePP, the index was used for class enumeration (15.22%), or researchers did not clarify for which purpose it was adopted (50%).

Only one study (Garnett et al., 2014) reported the mcaP_k_ and π_k_ to evaluate the classification accuracy of the LCA models. However, it did not report the 95% confidence interval of π_k_ to verify whether mcaP_k_ was included in it (Sorgente et al., 2019), nor calculate the discrepancy between π_k_ and mcaP_k_ (Schreiber, 2017). The classification error, also known as class error (e.g., Pandya, 2023) or classification uncertainty (e.g., Kyriakopoulos et al., 2015), was reported in 12 studies (3.80%). Only in half of the cases (50%) was it properly adopted to evaluate the classification accuracy. Only three studies (1%) adopted the OCC. In two of these studies, researchers used it properly (i.e., to evaluate classification accuracy).

Finally, the item-level classification diagnostics were rarely adopted. Only 18 studies (5.80%) verified whether item probabilities were > .70 and < .30 in order to evaluate class homogeneity. Furthermore, the estimated item endorsement odds ratio was adopted in just one study (Denson & Ing, 2014) to calculate class separation. Finally, we also found a paper that estimated class separation using Cohen’s *d* (Childs et al., 2023), and the authors justified this choice by referring to the paper by Tein et al. (2013).

Overall, regarding the classification diagnostics, we found that 68.4% of the studies (*n* = 214) reported at least one of these diagnostics (range 1–4). Unfortunately, only 60 of them (28.03%) adopted at least one of these classification diagnostics correctly, i.e., to evaluate the classification accuracy and not for class enumeration.

### Step 4: Selection of the LCA final model

Once the researchers have compared the fit indices of all the LCA solutions (class enumeration) and have more closely inspected the 1–3 best solutions, they should be ready to select the final LCA model based on all the evaluations reported above and describe the selected solution.

#### Current guidelines

Regarding the final solution, the guidelines suggest that researchers should include in the paper at least (1) a table in which the fit indices (and the classification diagnostics) are reported for the final solution as well as for all the other LCA models estimated (Aflaki et al., 2023; Schreiber, 2017; Weller et al., 2020), (2) the number or percentage of cases assigned to each class (Weller et al., 2020), and (3) a figure that represents the identified classes through item–response probability (Nylund-Gibson & Choi, 2018; Weller et al., 2020).

#### Review of psychology studies

Findings from our review suggest that most of the LCA studies in psychology report enough information about the final LCA solution. In particular, removing the 23 studies (7.3%) that did not report any fit indices or classification diagnostic for any of the LCA models they tested, the remaining 290 studies reported this information *at least* for the final model. Specifically, 86.2% of these 290 studies reported such information for both the final solution and the other LCA models, as guidelines suggest.

Across the 313 LCAs we reviewed, researchers selected as the final solution an LCA model that contained from just one class (i.e., Morandi et al., 2020) to 10 different classes (i.e., Iijima et al., 2020). The final LCA solution usually counts three or four classes (mode = 3; *Mdn* = 4; *M* = 3.68; *SD* = 1.20).

More than one-third of the reviewed studies (*n* = 126; 40.25%) did not report the entropy value for the selected solution. In the remaining studies we reviewed, the final solution had entropy of .80 on average (*M* = .80; *SD* = .10; *Mdn* = .81), with values ranging from .36 (Kukreti et al., 2022) to 1 (French et al., 2014). In 39.36% of the cases, it was lower than .80, i.e., the minimum recommended by many tutorials (Aflaki et al., 2023; Killian et al., 2019; Nylund-Gibson & Choi, 2018).

The information about the number or percentage of cases assigned to each class is the most commonly reported aspect of the final LCA solution. Indeed, across the reviewed studies, only 20 (6.39%) did not report this information. Finally, the use of a figure to represent the identified classes through the item–response probability plot is common in psychology. It was included in 194 studies out of 313 (61.98%). Of these, 48 (15.3% of 313) also reported item–response probability in tables. Finally, we found that 92 studies (29.4%) reported the item–response probability only in table format. The remaining 27 studies (8.6%) did not include any information about item–response probability. Overall, we found that 91.4% of reviewed studies provided a table or a graph of item–response probabilities, or both, for the indicators in each class.

Overall, the information related to the final solution is often reported in the manuscript (79.6% of the studies). In comparison, the remaining cases are reported in the Online Supplementary Materials (7.5%) or Appendix (10.4%), or they are partially reported in the manuscript and the Online Supplementary Materials (2.5%).

### Optional steps

Although the LCA can be considered concluded with the selection of the final model, across the tutorials we identified two additional steps that, although not mandatory, can be executed to provide more evidence and exploration of the LCA's final solution.

#### Validating the LCA model

##### **Current guidelines**

Researchers may want to validate the selected LCA model. Weller et al. (2020) specified that, although it is possible to publish LCA results without validation, this step is imperative for ensuring the typology is relevant to practice. Across the various tutorials, we identified two primary methods for validating the final LCA solution.

The first one involves demonstrating the LCA solution's reproducibility with new data, which can come from external datasets (Sinha et al., 2021) or can be a second subsample of the same larger sample from which the first tested subsample was extracted (split-sample cross-validation procedure; Masyn, 2013). In both cases, researchers aim to demonstrate that the solution found on the first dataset (or half of the sample) is the same as that found to work for the second set. There are two primary procedures for testing such reproducibility. Sinha et al. (2021) suggest performing all the LCA steps presented above (e.g., class enumeration, inspection of the best LCA solutions) for both datasets and verifying whether (1) the second dataset has the same number of classes as the best-fitting model in the first dataset, and if so, whether (2) the characteristics of the classes are similar to those identified in the primary model. Instead, Masyn (2013) proposes a stricter procedure: she suggests retaining all the model parameter estimates from the LCA model selected as the best model for the first half of the sample and then fitting the same LCA model to the second subsample, fixing all parameters to the estimated values retained from the first subsample. If the overall fit of this constrained model is acceptable, then the model is well validated.

The second proposed procedure to validate the selected LCA solution as the best one is to verify whether class assignments are related as expected to other variables (Petersen et al., 2019; Weller et al., 2020). Testing the association between a latent class variable and covariates is not always done to validate the LCA-selected model; it can also be done for different purposes (e.g., including control variables or exploring relationships). For this reason, we will present this as a separate step from the validation step (see the next section).

##### **Review of psychology studies**

Across the 313 studies we reviewed, we differentiated between studies that validated the final LCA model using two different datasets (as suggested by Sinha et al., 2021) and those using only one larger dataset divided into two or more subsets (as suggested by Masyn, 2013). We found four studies out of 313 (1.3%) testing the same LCA models on different datasets with a validation purpose, but only in two studies (Herzhoff & Tackett, 2016; Junus & Yip, 2022) were the two datasets fully equivalent (i.e., composed of the same kind of cases). In the other two cases (Beijers et al., 2019; Bianchi et al., 2017), these datasets were not entirely comparable. For example, Beijers et al. (2019) used as the first dataset a sample composed only of patients with depression or anxiety, while as the second dataset, the authors adopted a sample that included both patients and control subjects.

Regarding the split-half cross-validation procedure (Masyn, 2013), we identified 12 studies (3.83%) that divided the original sample into subsamples in order to validate the LCA final solution obtained from the first subsample. Except for Tang and Patrick (2020), who divided the original sample into 10 subsamples, all the other 11 studies divided the sample into two subsamples only. The division of the original sample into subsamples is done using a random procedure, as suggested by Masyn (2013). Only two studies did not specify how the subsamples were created (Gonsoulin et al., 2017; Holbrook & Hudziak, 2020). Finally, five out of these 12 studies (e.g., Denson & Ing, 2014) adopted exactly the validation procedure suggested by Masyn (i.e., testing an LCA model with constrained parameters on the second sample).

#### Including covariates

##### **Current guidelines**

Although it is not a mandatory step, many LCA tutorials (e.g., Nylund-Gibson & Choi, 2018; Weller et al., 2020) cite the potential inclusion of covariates and provide some information on this topic. Including covariates in the model consists of examining the antecedents of latent class membership (Nylund-Gibson & Choi, 2018), i.e., exploring whether class prevalence is equivalent across levels of a predictor (e.g., male vs. female). All the tutorials agree that this is a complex step, as many procedures exist to test the association between the latent class variable and covariates. Previously, researchers would directly include covariates in the same model used to identify the class solution (Vermunt, 2010). This one-step approach, however, can result in flawed, miss-specified models. Hence, in the last few years, it has been replaced with at least seven newer approaches (Nylund-Gibson & Choi, 2018). In these new approaches, the latent classes are generally estimated independently of the association between the latent class variable and covariates. Among these different approaches, tutorials (Nylund & Choi, 2018; Weller et al., 2020) agree that the current best practice consists of using either the three-step approach (Vermunt, 2010) or the Bolck, Croon, and Hagenaars (BCH) method (Bolck et al., 2004). Both of these methods require researchers to identify the measurement model (i.e., to select the best LCA model) and then add covariates. In the models with covariates, researchers fix the measurement parameters to those obtained in the model without covariates.

Finally, Ulbricht et al. (2018) and Killian et al. (2019) also presented another method to test the association between classes and covariates. It is the classify–analyze approach (referred to as the “*classic* three-step approach” in Killian et al., 2019), which consists of assigning individuals to their most likely class based on their greatest posterior probability of membership and then modeling the association between the assigned class and covariate of interest. In other words, the latent class variable is transformed into an observed variable where each case is modally assigned to just one class, ignoring the uncertainty of class membership.

##### **Review of psychology studies**

We found that 252 out of 313 LCA studies (80.5%) tested the association of the latent class variable with at least one covariate. We also aimed to classify the approach researchers adopted to test the association between latent classes and covariates. Considering that (1) specific methods to include covariates in an LCA model are many and are beyond the scope of this review, and that (2) reading the articles to review, we found that authors offer little information about how they included covariates in the model and which method they adopted, we decided to classify the selected procedure to test the association between latent classes and covariates using the same classification adopted in Ulbricht et al. (2018)’s review. Specifically, Ulbricht et al. (2018) distinguished the classify–analyze approach (i.e., the latent class variable is transformed into an observed variable, ignoring the uncertainty of class membership) from the model-based approaches (one-step and newer approaches—such as three-step and BCH, where the actual probability of a covariate conditional on latent class membership is modeled). In the Ulbricht et al. (2018) review, the most frequently reported approach was the classify–analyze approach. Instead, we found a more equal distribution between the two approaches. Seventy-two studies (49.7%) adopted one of the model-based approaches, while 73 studies (50.3%) adopted the classify–analyze approach. From this comparison, we excluded 107 LCA studies (42.43% of the 252) that, even if they tested the association between classes and covariates, did not provide enough information about how this association was tested to classify them into one of the two approaches.

## Discussion

The purpose of this systematic review was to document the use of LCA in psychology, mapping both the prevalence and quality of LCA studies. Across various databases (PsycInfo, Scopus, Psychology Database, Web of Science, and JSTOR), we identified 7,580 peer-reviewed records that mentioned “latent class analysis” in their abstracts. After removing duplicates (*n* = 3,809) and randomly extracting a representative subsample from the 3,771 remaining records, 377 documents were evaluated for eligibility. Of those, 251 records (for a total of 313 studies) satisfied both inclusion and exclusion criteria and were reviewed for the current study.

First, in terms of prevalence, we found that the number of LCA studies published in psychology has increased exponentially over the past decade. Similar trends were observed for studies using latent variable mixture methods in general (Killian et al., 2019), as well as for the adoption of LCA for the study of depression (Ulbricht et al., 2018), mental health (Petersen et al., 2019), or healthcare and public health (Zhou et al., 2018). Our review is the first to broadly investigate the use of LCA in the psychology disciplines without focusing on a specific construct or topic. We found that most LCA studies were published in psychology journals related to the topic of illness and mental health (e.g., *Child Abuse & Neglect*, *Journal of Affective Disorders*, *Drug and Alcohol Dependence*), suggesting that interest in identifying different classes of individuals is prominent in clinical psychology. We could speculate that this reflects the greater interest in classification among clinical psychologists due to their habit of using diagnosis.

Findings from our review indicate that psychology has already made significant progress in the use of LCA. In fact, the number of publications per year has quadrupled compared to a decade ago. However, it appears that neither authors nor peer reviewers are yet fully aware of the methodological standards that LCA studies should meet, and “further work is needed to improve the rigor with which the method is applied” (Petersen et al., 2019, p. 14).

Specifically, we identified three main issues. First, many papers contained methodological errors—for example, failing to include the one-class solution in the set of models tested or using classification diagnostics such as entropy to compare model solutions. Second, crucial information was frequently missing, such as the estimation method used or how missing data were handled. Third, the same indices or procedures (e.g., SABIC vs. SSBIC) were often labeled inconsistently across studies, leading to confusion.

### Best practices and recommendations

To help address these issues, we provide concise guidelines for each step of conducting an LCA. These guidelines integrate best practices from existing tutorials, resolve common inconsistencies, incorporate recent developments, and specify what should be done, what information should be reported, and how it should be done.

#### Step 1: LCA methodological decisions

Before running an LCA, researchers should carefully consider the following decisions:

- **Sample**: When defining the sample *size*, we recommend using the heuristic of 500 cases as a general guideline. However, for simpler LCA models with a few well-separated classes, a smaller sample may be sufficient. Importantly, researchers should remember that a large but non-*representative* sample can also lead to misleading results. Both the sample size and the sampling method should be clearly reported in the paper.
- **Indicators**. Variables adopted as observed indicators of the LCA should be clearly reported in the “Measures” section of the paper, along with the response options for each indicator. Furthermore, when selecting observed indicators to identify latent classes, researchers should simultaneously consider the following aspects:

- *Literature*. Researchers should consult the existing literature on their topic of interest. Prior studies using LCA can help identify suitable indicators for this purpose. It is important to select indicators that comprehensively capture the phenomenon and are distinct from one another, avoiding redundancy.
- *Number of indicators*. While including more indicators may seem advantageous, the decision should be grounded in theoretical considerations. A smaller set of meaningful, nonoverlapping indicators is preferable to a large set of redundant ones.
- *Measurement scale. *Researchers must decide how each indicator will be measured. While LCA can accommodate continuous and categorical variables in the same model, dichotomous indicators are often easier to interpret than polytomous indicators and can facilitate the assessment of class homogeneity and separation (Masyn, 2013). However, dichotomization should only be applied when two response categories are sufficient to meaningfully represent the variable and address the research question.
- *Response frequencies*. After selecting the indicators, researchers should revisit their choices through preliminary data analysis. For instance, if a dichotomous indicator shows minimal variance (e.g., only 0.8% of participants responding “yes”), it should be excluded from the analysis despite its theoretical relevance, as it will not contribute meaningfully to class differentiation. Similarly, if a response category of an ordinal indicator is selected by fewer than 10% of participants, it may be appropriate to collapse it into an adjacent category. Nonetheless, frequency-based decisions should always be balanced with theoretical relevance. For example, a rare indicator may still be essential if it helps identify a specific, infrequent condition (e.g., rare diagnosis).

- **Software**. For a complete list of software available to run an LCA and their characteristics, we suggest that readers check Table S2 of Aflaki et al. (2023). We would also add to this list less common software, such as Sawtooth Software, WinMira, and LEM—which we found in our review—as well as Jamovi, a free and open statistical platform that is intuitive to use and built on top of the R statistical language (The jamovi project, 2023) and that recently added a package to run LCA. For simple LCA models, there will not be significant differences across software packages; this choice is more relevant when researchers are estimating more complex mixture models, using different estimation techniques, or seeking specific graphical output (Nylund-Gibson & Choi, 2018). Researchers should specify both the software and its version used to perform the LCA in the paper. Additionally, if they investigated associations between latent classes and external variables using the classify–analyze approach, they should clearly state which software was used for these follow-up analyses.
- **Estimation method**. When selecting estimation methods, researchers should consider that the choice of estimator may depend on several criteria, such as sample size, number of variables, computational speed, and the management of missing data (for details, see B. O. Muthén et al., 2015). We agree with Weller et al. (2020) that researchers new to LCA should use the default option until they begin to conduct more advanced LCA models. However, researchers should at least specify in their report that they have used such default options rather than not offering any information about estimator methods and random starting values. Regarding the selection of “random starting values,” we suggest that researchers first determine the default values set by the software they have adopted (e.g., in Mplus, the default is 20 random sets of starting values for the initial stage and four optimizations for the final stage; L. K. Muthén & Muthén, 1998–2017), and second, increase those values if they are lower than what is suggested (e.g., the first value should be at least 50–100, according to Masyn, 2013). Furthermore, those values can be further increased when the output file has clues that the “best” solution found is not the true maximum likelihood solution (e.g., a low frequency of replication of the apparent global solution across the sets of random starting values; see Masyn, 2013, for more details).
- **Missing data**. We believe that, before deciding which missing method to adopt (e.g., deletion, multiple imputation, FIML), researchers should investigate the mechanism of their missing data. If the mechanism is MCAR or MAR, we suggest adopting FIML as it is the default method and does not require an additional data imputation step to the analysis. In the case where missing data are not missing at random (NMAR), the LCA will produce biased parameters. There is no recommended way to address NMAR. However, we believe researchers should at least report in their article that their analysis is based on this type of missingness. Regarding FIML as it is the default method in many software programs, it is worth noting that it is utilized to account for missing data only in unconditional LCA models (i.e., models without covariates). Instead, listwise deletion is automatically used for missing data on the covariates when classes are regressed on covariates (Caba et al., 2022). Overall, in addition to reporting the method used to handle missing data (e.g., FIML), researchers should always report both the amount of missing data and the underlying missing data mechanism, as both may affect the accuracy of the results (Swanson et al., 2012; Wolf et al., 2013).

#### Step 2: Class enumeration

Once the researcher has made all the decisions outlined above, they should begin class enumeration by specifying a one-class LCA model and then fitting additional models, incrementing the number of classes by one.

Regarding stopping rules, we believe all those proposed across various tutorials (e.g., non-identified models, worsening model fit, or small class sizes) should be considered when deciding when to stop estimating additional models. However, these considerations are relevant primarily when researchers adopt an *exploratory* approach. As highlighted by Sorgente et al. (2019), mixture models can be estimated using either an exploratory or a confirmatory approach. When the LCA is guided by a theory that specifies the expected number of latent classes, a *confirmatory* approach may be used (Wang & Hanges, 2011).

For example, if a researcher is investigating the cognitive development of children aged 1–13 based on Piagetian theory (1973), four developmental stages—and thus four latent classes—are expected: the sensorimotor stage (birth to age 2), preoperational stage (ages 2–7), concrete operational stage (ages 7–11), and formal operational stage (age >11). In such a case, the goal is to confirm the four-class model, so it is not necessary to estimate every possible solution. Researchers could instead estimate the four-class model and compare its fit with that of adjacent models (e.g., three- and five-class solutions) to demonstrate that the four-class model provides the best representation of the data.

Once all LCA models have been estimated, researchers should compare them using absolute, inferential relative, and descriptive relative fit indices.

- **Absolute fit indices** (e.g., likelihood ratio chi-square goodness-of-fit test and standardized residuals) should be checked for each model. Evaluations based solely on relative fit indices do not provide information about the absolute adequacy of the models; one model may fit better than another, yet both may still provide a poor overall fit (Masyn, 2013). We also recommend referring to the likelihood ratio chi-square test using the notation ${\chi }_{LR}^{2}$ to reduce the inconsistency found in the literature (${\chi }_{LR}^{2}$ , ${\chi }_{LRT}^{2}$ , *G*^2^ , *L*^2^).
- **Inferential relative fit indices** such as the adjusted Vuong–Lo–Mendell–Rubin likelihood ratio test (adjusted VLMR) and the bootstrap likelihood ratio test (BLRT) should be adopted. Researchers should avoid reporting only the value of these tests (e.g., Fu & Vaughn, 2016); instead, they should always report the *p*-values, preferably along with other output from the software (e.g., difference across the two compared models in terms of log-likelihood and number of parameters), as the *p*-value is the key element for interpretation. It is worth noting that sometimes adjusted VLMR and BLRT will not yield nonsignificant *p*-values even when model improvements become negligible. In such cases, researchers may plot the log-likelihood values against the number of classes and look for an “elbow” in the curve—i.e., a point where the increase in model fit plateaus. The model corresponding to that elbow should be considered optimal (Masyn, 2013).
- **Information-criterion-based comparisons of relative fit** should include all indices provided by the chosen software. If any are unavailable, researchers can compute them using formulas from Nylund-Gibson & Choi (2018, see Table 1). We suggest reporting all commonly used indices—AIC, CAIC, BIC, SABIC, and AWE—and weighting them appropriately depending on the analytical conditions (e.g., sample size) that make a difference, as indicated by simulation studies (Morgan, 2015; Whittaker & Miller, 2021; Yang, 2006). To ensure consistency, we recommend using the acronym SABIC to refer to the sample-size-adjusted BIC, as various alternative acronyms were observed in the literature (e.g., SSBIC, NBIC, adjustedBIC). Finally, the evaluation of information criteria should be integrated with the use of the approximate Bayes factor (BF) and approximate correct model probability (cmP). These provide insight into how much better a given model is compared to another or relative to a set of competing models (Masyn, 2013).

#### Step 3: Closer inspection of the best solution(s)

Based on the results of Step 2, researchers should have identified between one and three plausible solutions and should now examine them more closely to determine the best-fitting model. Even when a single solution appears most plausible based on fit indices, it is essential to assess whether that solution also demonstrates sufficient quality. Indeed, a mixture model may exhibit a good overall fit but still yield poor latent class assignment accuracy. The quality of the selected solution(s) should be evaluated based on two main criteria: interpretability and classification diagnostics.

Regarding *interpretability*, researchers should examine the substantive meaning of the classes and assess whether the solution is theoretically and empirically sound. This assessment should consider various aspects such as consistency with theoretical expectations, class size, model parsimony, and the presence of the “salsa effect.” To enhance transparency and rigor, researchers are encouraged to explicitly state the criteria they used to evaluate interpretability. Additionally, interpretability can be strengthened if researchers formulate hypotheses in advance about the expected latent classes—both in terms of response patterns and relative sample sizes—based on theoretical or empirical grounds (Morgan, 2015). Once a solution is selected, researchers can then assess the degree to which the identified classes align with these expectations.

Regarding *classification diagnostics*, researchers should evaluate class homogeneity and separation by calculating and reporting all relevant indices. These include entropy, average posterior probabilities (AvePP), modal class assignment proportions (mcaP_k_ and π_k_), odds of correct classification (OCC), item–response probability cutoffs, and estimated item endorsement odds ratios (see Masyn, 2013). Importantly, these diagnostics should *not* be used to determine the number of classes (i.e., class enumeration) but rather to assess the quality and reliability of the chosen solution(s).

#### Step 4: Selection of the LCA final model

Once researchers have selected the final LCA model based on the evaluations conducted in Step 3, they should proceed to describe this solution in detail. First, a table should be presented that reports the fit indices for the final model alongside the corresponding indices for all other estimated LCA models (see Step 2). In addition, we recommend including a separate table reporting classification diagnostics (see Step 3), but only for the one to three best-fitting solutions, as exemplified by Garnett et al. (2014).

When reporting the distribution of participants across classes, researchers should include both the absolute number and the percentage of cases assigned to each class to enhance interpretability and clarity.

With regard to item–response probabilities, we believe that these do not necessarily need to be presented in a figure. Graphical representations are particularly useful when all indicators have the same response scale and when the number of response categories is small (e.g., dichotomous items). In cases where response formats vary or include more categories, a table may provide a clearer and more detailed description of item–response probabilities than a figure.

Finally, when opting for a graphical representation, researchers may choose between line graphs and bar charts, both of which are widely used. However, we encourage consideration of the alternative and more informative visualization approaches proposed by Van Lissa et al. (2023).

#### Optional steps

##### **Validating the LCA model**

Although the LCA procedure can be considered complete after Step 4, when researchers have sufficient resources (e.g., access to a second dataset or a large enough sample to be split into two subsamples), we recommend conducting a model validation. It is important to emphasize that a straight validation (Howitt & Cramer, 2020) requires the two datasets (or subsamples) to be composed of equivalent cases—that is, samples that are comparable in key characteristics.

We encourage researchers to follow the validation approach proposed by Masyn (2013), which consists of testing the best-fitting model identified in the first dataset/sample on the second one by constraining its parameters and evaluating model fit. This approach is more efficient and allows for a more stringent test of model generalizability than the method proposed by Sinha et al. (2021), which involves re-estimating all possible LCA models on the second dataset or sample.

##### **Including covariates**

Researchers may also wish to examine the association between latent class membership and external variables. If so, we recommend clearly stating the rationale for including covariates, specifying one of the following purposes:

- *Validation of the LCA model*: As described above, exploring how latent classes relate to external variables can serve as a form of model validation. In this case, researchers should ideally formulate hypotheses about expected associations. According to Petersen et al. (2019), it may be sufficient to state that at least one class is expected to show a different relationship with the covariate compared to others.
- *Control variables*: Covariates can be included to control for potential confounding factors in estimating class membership and sample heterogeneity (Killian et al., 2019).
- *Exploratory purposes*: Researchers may be interested in exploring how latent classes differ across external variables, for instance, by identifying whether certain classes are more prevalent among specific demographic groups (e.g., a higher likelihood of class membership among women than among men).

Once the rationale is defined, the appropriate method for assessing covariate effects must be selected. In line with Nylund-Gibson & Choi (2018) and Weller et al. (2020), we recommend using modern approaches such as the three-step approach or the Bolck–Croon–Hagenaars (BCH) approach and avoiding the older one-step approach, which has limitations in terms of interpretability and flexibility.

While generally discouraged, the classify–analyze approach may be considered when the LCA model demonstrates strong classification accuracy (e.g., entropy > .80; Clark & Muthén, 2009). In such cases, assigning individuals to classes based on their most likely class membership may be acceptable, particularly when subsequent analyses are too complex to perform within the LCA framework.

Regardless of the method chosen, we urge researchers to be as transparent and precise as possible in describing how they tested the association between latent classes and covariates. This includes (1) using consistent terminology (e.g., avoiding confusion between “classify–analyze” and “classical three-step” when referring to the same method), (2) clearly specifying the analytical strategy used, keeping in mind that implementations of these methods may vary across software packages (Weller et al., 2020), and (3) indicating whether multiple covariates were included simultaneously in the model or tested individually.

For a detailed overview of best practices in this area, we recommend consulting key references on this topic (e.g., Asparouhov & Muthén, 2014; Nylund-Gibson & Choi, 2018; Nylund-Gibson & Masyn, 2016; Vermunt & Magidson, 2016).

### Limitations and future studies

We acknowledge that the present systematic review has limitations, a fact that has been highlighted in similar reviews (e.g., Killian et al., 2019). In particular, our review is not fully comprehensive, as we omitted non-English articles, included only peer-reviewed studies published in the last 10 years, and searched for the expression “latent class analysis” in the abstract only. Moreover, only 10% of the records in the sample were extracted and coded when they met the eligibility criteria. Nevertheless, we consider our review to be a significant contribution to the representation of LCA use in psychology, given that we reviewed 251 articles (presenting 313 different LCA studies). In previous reviews of LCA, the number of studies included has ranged from 23 (Petersen et al., 2019) to 99 (Zhou et al., 2018).

To those engaged in research activities pertaining to LCA and systematic reviews, we propose that a fruitful avenue for further investigation would be to conduct the following systematic reviews. Firstly, we posit that a systematic review of the utilization of LPA in the field of psychology may prove to be of significant interest. We decided not to include both LCA and LPA in this review, given that specific details of the analysis differ between the two methods.

Second, we suggest that a systematic review be conducted that focuses specifically on the inclusion of covariates in LCA models, providing sufficient space for the different methods available for testing the association between class membership and other variables. This would enable us to better understand the factors that limit researchers’ ability to accurately report how they have carried out this step of the analysis. Furthermore, such a review could also examine whether the inclusion of covariates in the model is conducted in a manner similar to the inclusion of (distal) outcomes, i.e., variables that are expected to be influenced by class membership (Nylund-Gibson & Choi, 2018).

Finally, we believe that it would be interesting to conduct a new systematic review of LCA studies in psychology in 5–10 years. We believe that higher-quality studies will be identified. Indeed, the difference we found in the current review between tutorial guidelines and research practice may reflect the fact that most LCA tutorials have been published in the last 3–4 years (Aflaki et al., 2023; Sinha et al., 2021; Weller et al., 2020). We can expect the impact of such tutorials, including the current review and its suggestions, to blossom over the next few years.

## Conclusion

The current study is a systematic review that broadly examines the application of LCA in psychology, with a particular focus on the methodological and statistical characteristics of studies that have employed this analysis. More than 300 studies (presented in 251 publications) were coded, and information on how they carried out each step of LCA was retrieved. For each step, the current review indicated what published tutorials suggest should be done (*Current guidelines*) and what the reviewed studies actually did (*Review of psychology studies*). The results suggest that LCA publications in psychology still lack rigor. Improvements are needed in (1) reducing the number of methodological errors made by researchers in the various steps leading to the identification of the best LCA solution, (2) improving the amount and quality of information provided about how the LCA was conducted and the results obtained, and (3) increasing the consistency of LCA studies in terms of form and content.

To help address these issues, the “Best practices and recommendations” section provides concise guidelines for each step in conducting an LCA, specifying what should be done, what information should be reported, and how to do it.

We anticipate that this systematic review of LCA studies in psychology will be a valuable resource for both psychology researchers who wish to conduct rigorous LCA studies and those involved in the peer review of such studies.

## Data Availability

Data and research materials are available at https://osf.io/26xps/files/osfstorage.

## Code availability

Analysis code is available at https://osf.io/26xps/files/osfstorage.
