# Publisher Correction: A systematic review of latent class analysis in psychology: Examining the gap between guidelines and research practice

**DOI:** 10.3758/s13428-025-02871-4

**Published:** 2025-11-03

**Authors:** Angela Sorgente, Rossella Caliciuri, Matteo Robba, Margherita Lanz, Bruno D. Zumbo

**Affiliations:** 1 Department of Psychology, Università Cattolica del Sacro Cuore, Largo Gemelli, 1–20123, Milan, MI Italy; 2 University of British Colombia, Vancouver, Canada

**Publisher Correction: Behavior Research Methods (2025) 57:301**

10.3758/s13428-025-02812-1

The original online version of this article was revised: Under the heading Missing Data, the following text was inadvertently deleted:

Current Guidelines. According to Sinha et al. (2021), three methods are widely used to deal with missing variables when performing LCA: deletion, multiple imputation, and full information maximum likelihood (FIML). The first method includes only complete cases in the analysis, but this implies that large swathes of data can be lost and that a large portion of participants is not represented anymore in the final sample. In general, this is the least preferred of the three methods and should seldom be used (Sinha et al., 2021). In relation to LCA, the other two methods have their inherent advantages and disadvantages. Both approaches work on the assumption that the data are either missing completely at random (MCAR) or missing at random (MAR) (Masyn, 2013). The ML (and MLR) estimators automatically adopt the FIML method to manage missing data, i.e., individual cases are not excluded from the analysis unless they are missing data on all the observed items. Aflaki et al. (2023) also report a solution different from the three reported above (i.e., deletion, multiple imputation, and FIML). They suggested treating “variables containing missing values as categorical variables, where missing values are treated as bring in their own category” (p. 171).

Review of Psychology Studies. Results of our review indicate that almost all the studies (82.7%) did not report information about the method they adopted to manage missing data. Among the 54 studies that reported information about the method they adopted to manage missing data, most (81.48%) adopted the FIML, which is in agreement with the finding from Killian et al. (2019). Other studies reported handling missing data within the expectation-maximization algorithm (0.55%) and listwise deletion (0.74%). Only two studies reported the use of multiple imputation (Bayly et al., 2022; Yuan et al., 2022), and one study reported the use of the Maximum Likelihood with Missing Values (MLMV; Harrison et al., 2022) method.

The text has been restored to the article.

## Data Availability

Data and research materials are available at https://osf.io/26xps/overview

